# Simultaneous infestations in dogs by different tick species (Acari: Ixodidae) in two areas of the Western Brazilian Amazon

**DOI:** 10.1590/S1984-29612025036

**Published:** 2025-06-02

**Authors:** Ivaneide Nunes da Costa, Marcos Valério Garcia, Vanessa Paiva dos Santos, Natalia Vitória Coelho Costa, Angélica Lorena Pereira Mendes Carioca, Renato Andreotti, Jansen Fernandes Medeiros, André de Abreu Rangel Aguirre

**Affiliations:** 1 Laboratório de Entomologia, Fundação Oswaldo Cruz – Fiocruz Rondônia, Porto Velho, RO, Brasil; 2 Programa de Pós-graduação em Biologia Parasitária, Instituto Oswaldo Cruz, Fundação Oswaldo Cruz – Fiocruz Rondônia, Porto Velho, RO, Brasil; 3 Laboratório de Biologia do Carrapato, Embrapa Gado de Corte, Campo Grande, MS, Brasil; 4 Programa de Pós-graduação em Biologia Experimental, Universidade Federal de Rondônia, Porto Velho, RO, Brasil; 5 Laboratórios de Biologia e Biologia Molecular do Carrapato, Embrapa Gado de Corte, Campo Grande, MS, Brasil; 6 Plataforma de Criação e Experimentação Animal, Fundação Oswaldo Cruz – Fiocruz Rondônia, Porto Velho, RO, Brasil

**Keywords:** Dogs, ticks, Amazon, Cães, carrapatos, Amazônia

## Abstract

Although there are reports on many species of ticks infesting dogs, including from the Amazon region, simultaneous infestations by different species are poorly discussed in the literature. Therefore, this study aimed to report simultaneous infestations in dogs by different species of ticks, involving up to six species, in two areas of the western Brazilian Amazon, specifically in the border region between the states of Rondônia and Amazonas, in northern Brazil. Ticks were collected between 2021 and 2023 from dogs on private properties surrounding two conservation units: Municipal Natural Park of Porto Velho (NP), and Mapinguari National Park (MNP). The following species were identified: *Amblyomma ovale*, *Amblyomma scalpturatum*, *Amblyomma latepunctatum*, *Amblyomma oblongogutattum*, *Amblyomma coelebs*, *Amblyomma naponense* and *Rhipicephalus sanguineus* s.l.. Among the 342 dogs sampled, 184 were from the area around the NP and 158 were from the area around the MNP. A total of 34 dogs (9.9%) exhibited simultaneous tick species infestations, ranging from two to six tick species; 26 dogs (16.5%) with simultaneous infestations were from the MNP area, and eight dogs (4.3%) were from the NP area. This study is the first to report the simultaneous occurrence of four, five or six tick species on individual dogs.

## Resumo

Embora existam relatos de diversas espécies de carrapatos parasitando cães, principalmente na região Amazônica, relatos de parasitismo simultâneos de carrapatos em animais é pouco discutido na literatura. Diante do exposto, o presente estudo relata a presença de cães com infestações simultâneas de até seis espécies diferentes de carrapatos em duas áreas da região Amazônica, zona de fronteira entre Rondônia e Amazonas. Os cães amostrados eram oriundos de propriedades particulares adjacentes aos Parques Nacional Mapinguari (PNM) e Natural Municipal de Porto Velho (PN), Rondônia. As seguintes espécies de carrapatos ninfas e adultos foram identificados: *Amblyomma ovale*, *Amblyomma scalpturatum*, *Amblyomma latepunctatum*, *Amblyomma oblongogutattum*, *Amblyomma coelebs*, *Amblyomma naponense* e *Rhipicephalus sanguineus* s.l., utilizando chaves dicotômicas para ninfas e adultos. Foram abordados 342 cães, sendo 158 oriundos do PNM e 184 do PN. Um total de 34 (9,9%) cães estavam com infestações simultâneas por duas até seis espécies de carrapatos por animal, sendo 26 (16,5%) dos entornos do PNM e oito (4,3%) do PN. Este estudo relata pela primeira vez a infestação simultânea por quatro, cinco ou seis espécies de carrapatos em cães individuais.

## Introduction

Dogs are primary hosts for ticks *Rhipicephalus sanguineus* sensu lato (s.l.) complex, commonly known as the “brown dog tick,” which are found worldwide. *Rhipicephalus linnaei* is the most commonly found tick of this complex in Brazil, a tropical country, while *R. sanguineus* sensu stricto (s.s.) is found in temperate areas in the southern regions of the country (Labruna & Pereira, 2001; Slapeta et al., 2022). These tick species were probably introduced to Brazil from Africa and Europe, respectively, and have adapted to the Brazilian climate (Nava et al., 2015; Slapeta et al., 2022). Additionally, other tick species of the Brazilian fauna have been found parasitizing dogs in the northern, southern, southeastern and northeastern regions of the country (Aragão, 1936; Freire, 1972; Labruna et al., 2000, 2001, 2007; Martins et al., 2009; Costa et al., 2020, 2021).

Dogs that are reared loose and have free access to native vegetation are positively associated with parasitism by tick species and may acquire pathogens from the enzootic cycle (Cunha et al., 2014). Some studies indicate that rural dogs play an important role in the epidemiological cycle of certain tick-borne diseases due to the proximity of infected ticks to domestic environments, thereby increasing the likelihood of human contact with these ticks, as well harming the health of wildlife by carrying parasites and associated infectious agents (Gordon et al., 1984; Paddock et al., 2002).

Given the significance of dogs in carrying infected ticks in Brazil and the limited discussion on simultaneous infestations of tick species on dogs, this study aimed to report simultaneous tick infestations on rural dogs from two areas of the western Brazilian Amazon. These findings may contribute to the literature on epidemiological studies of dog-associated tick-borne diseases, particularly in the Amazon region, a vast biome with limited knowledge about the epidemiology of these diseases.

## Materials and Methods

### Study area

**Figure 1:**
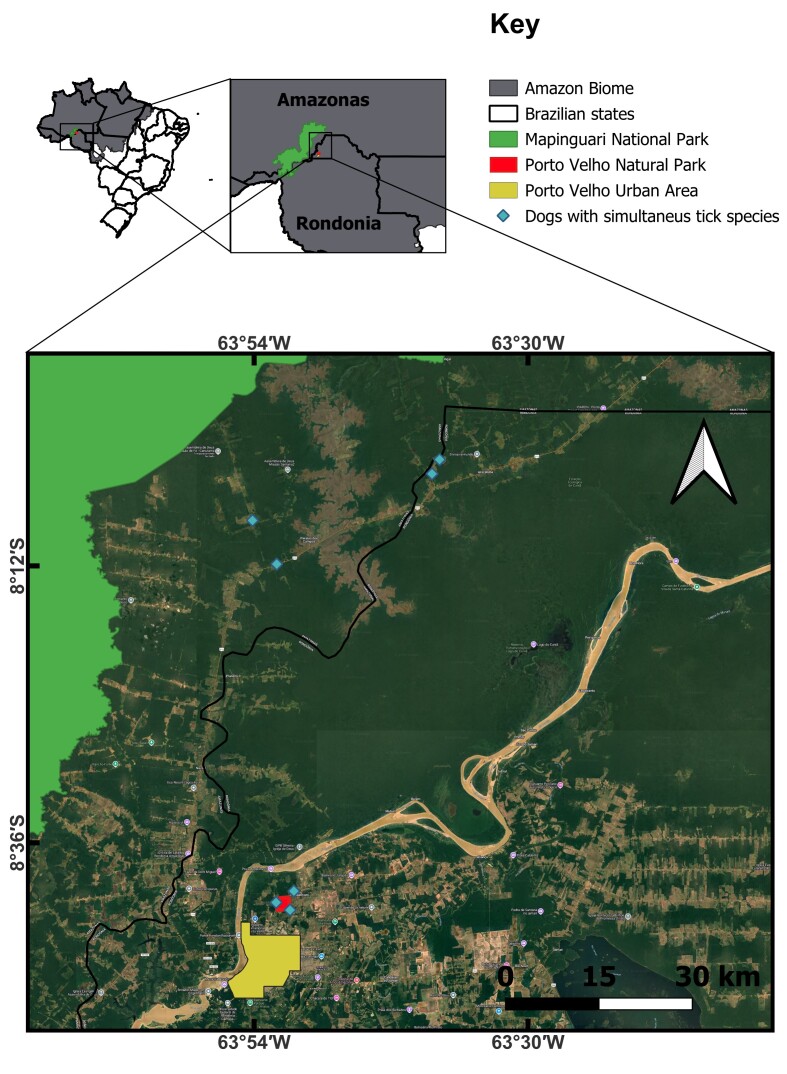
Map of the study area. The red area represents the Municipal Natural Park of Porto Velho (NP) and the green area is the Mapinguari National Park (MNP). The blue rhombuses represent properties visited during the study period that have dogs with simultaneous parasitism of different tick species, which include the surroundings of the NP and along the BR-319 highway and its branches nearby to MNP.

This study was conducted on private properties in the surrounding areas of two conservation units (CUs) located in a border region between the northern Brazilian states of Rondônia and Amazonas, which are part of the Amazon rainforest: Municipal Natural Park of Porto Velho (NP), and Mapinguari National Park (MNP) (Figure 1).

Natural Park is a municipal CU located in the urban area of the municipality of Porto Velho and covers 390.8 hectares (ha). The main entrance is located at 8°41’11.34” S and 63°52’4.73” W. The private properties included in this study were in the neighborhoods/districts of Belmont, Nova Esperança, and Terra Santa, which are located in the surrounding areas of the park (Figure 1).

Mapinguari National Park is a federal CU of 1,776,915.16 ha and encompasses the municipalities of Canutama and Lábrea (Amazonas state) and Porto Velho (Rondônia state). The private properties included in this study were situated along a 30 km stretch of the BR-319 highway, between highway markers 70 and 100, on the route from Porto Velho to Humaitá (Amazonas), and three dirt roads branching off the BR-319, known as: Ramal km 70, Ramal Mapinguari, and Ramal São Bernardo (Figure 1). These properties share forest areas that are connected to the MNP.

### Tick collections from dogs and taxonomic identification

Ticks were collected from the dogs in September 2021, March 2022, September 2022, and March 2023, with each sampling period spanning three days at each location. These months were chosen so as to include collections in the rainy (March) and the dry (September) seasons for the region. The selection of sampled dogs was based on convenience, prioritizing properties with free-ranging dogs that had access to forested areas connected to each CU. The dogs were sampled with the owners’ authorization, after they signed an informed consent form. The study was conducted in accordance with the guidelines of the National Animal Experimentation Control Council (CONCEA) and approved by the Ethics Committee of Fiocruz Rondônia (CEUA) under protocol number 2019/21.

The dogs were restrained with the help of their owners, and each dog was inspected for ticks during five minutes. Flat ticks and engorged larvae and adults were placed in 1.5 mL microtubes containing isopropanol and the tubes were labeled with a code corresponding to the dog. Engorged nymphs and one adult were kept alive in vials and maintained in a chamber with 80-85% humidity, temperatures of 23-29 °C, and a 12/12-hour light/dark cycle to allow molting and oviposition.

Taxonomic identification of the ticks was performed using a stereomicroscope and dichotomous keys for the family and genera of the Ixodidae family, as well as for nymphs and adults of species of the genus *Amblyomma* (Onofrio et al., 2006; Martins et al., 2010; Dantas-Torres et al., 2019). Larvae were molecularly identified via the sequencing of a 16S RNA gene fragment (Mangold et al., 1998). DNA was extracted using the guanidine isothiocyanate-phenol method, as described elsewhere (Sangioni et al., 2005).

## Results

A total of 342 dogs were sampled in this study, with 184 from the surrounding areas of NP and 158 from the surrounding areas of MNP. A total of 100 dogs were found to have ticks, and 34 of them presented simultaneous infestations of at least two tick species. In the NP area, 39/184 dogs (21,2%) were infested with ticks, eight dogs (4.3%) exhibited simultaneous infestations with two tick species. Three tick species were identified on dogs in the NP area: *Rhipicephalus sanguineus* s.l. (128 females, 154 males, and 42 nymphs), *Amblyomma ovale* (three females and 14 males), *Amblyomma coelebs* (one nymph), and *Amblyomma* sp. (two nymphs). In the MNP area, 61/158 dogs (38.6%) were found to have ticks, with 26 dogs (16.5%) presenting simultaneous infestations involving up to six tick species. A total of seven tick species were identified on dogs in the MNP area: *R. sanguineus* s.l. (one nymph, 118 females and 107 males), *Amblyomma oblongoguttatum* (14 nymphs, six females and 15 males), *A. ovale* (two nymph, 24 females and 35 males), *A. coelebs* (five nymphs and two larvae), *A. scalpturatum* (21 nymphs), *Amblyomma naponense* (one nymph), *Amblyomma latepunctatum* (one nymph) and *Amblyomma* sp. (30 larvae and 13 nymphs) (Table 1).

**Table 1:** Simultaneous and individuals’ infestations of dogs by ticks of the genera *Amblyomma* and *Rhipicephalus* in the surrounding areas of the Municipal Natural Park of Porto Velho (NP) and the Mapinguari National Park (MNP).

**Ticks**	**Nº of infested dogs (%)**		**Total (%)**
	**Natural Park**	**Mapinguari National Park**	
*R. sanguineus* s.l. + *A. ovale*	6 (3.3)	7 (4.4)	13 (3.8)
*A. ovale* + *A. scalpturatum* + *A. oblongoguttatum*	0 (0.0)	4 (2.5)	4 (1.2)
*Amblyomma* sp. + *A. oblongoguttatum*	0 (0.0)	1 (0.6)	1 (0.3)
*R. sanguineus* s.l. + *A. ovale* + *A. scalpturatum* + *A. oblongoguttatum*	0 (0.0)	6 (3.8)	6 (1.8)
*R. sanguineus* s.l. + *A. ovale* + *A. oblongoguttatum*	0 (0.0)	1 (0.6)	1 (0.3)
*A. coelebs* + *A. ovale* + *A. latepunctatum* + *A. scalpturatum* + *A. oblongoguttatum*	0 (0.0)	1 (0.6)	1 (0.3)
*R. sanguineus* s.l. + *A. oblongoguttatum*	0 (0.0)	1 (0.6)	1 (0.3)
*R. sanguineus* s.l. + *A. ovale* + *A. scalpturatum* + *A. oblongoguttatum* + *A. coelebs*	0 (0.0)	1 (0.6)	1 (0.3)
*R.sanguineus* s.l. + *A.ovale* + *A. scalpturatum* + *A. oblongoguttatum* + *A. coelebs* + *A. naponense*	0 (0.0)	1 (0.6)	1 (0.3)
*A. ovale* + *A. oblongoguttatum*	0 (0.0)	1 (0.6)	1 (0.3)
*R. sanguineus* s.l. + *A. ovale* + *A. scalpturatum*	0 (0.0)	1 (0.6)	1 (0.3)
*R. sanguineus* s.l. + *A. scalpturatum*	0 (0.0)	1 (0.6)	1 (0.3)
*R. sanguineus* s.l .+ *Amblyomma* sp.	1 (0.5)	0 (0.0)	1 (0.3)
*Amblyomma* sp. + *A. ovale*	1 (0.5)	0 (0.0)	1 (0.3)
*R. sanguineus* s.l.	29(15.8)	26 (16.5)	55 (16.1)
*A. ovale*	2 (1.1)	3 (1.9)	5 (1.5)
*A. oblongoguttatum*	0 (0.0)	5 (3.2)	5 (1.5)
*A. scalpturatum*	0 (0.0)	1 (0.6)	1 (0.3)

Of the ticks mentioned above, some were collected engorged from the dogs, and some of them could be identified after molting. The engorged ticks from dogs of the NP area were two *A. ovale* females, four *A. oblongoguttatum* females and one *A. coelebs* nymph and two. nymphs of the genus *Amblyomma*. The engorged ticks from the dogs of the MNP area were nine *A. ovale* females, one *A. oblongoguttatum* females, five *A. oblongoguttatum* nymphs and 10 larvae of the genus *Amblyomma*. Of the five engorged *A. oblongoguttatum* nymphs collected in the MNP area, three molted into adults (two females and one male). In addition, one engorged *A. ovale* female laid eggs. Two engorged *A. coelebs* larvae from two dogs of the MNP area were molecularly identified by sequencing a partial 16S rRNA gene fragment, with sequences presenting 99.76% identity and 99% query cover for *A. coelebs* in the GenBank (accession numbers MH513258 and KU001160).

## Discussion

Free-ranging domestic animals that come into contact with wild environments may experience simultaneous infestations of tick species, thereby becoming accidental hosts to the native tick fauna and thus susceptible to pathogen infections from the sylvatic environment (Szabó et al., 2001). The phenomenon of simultaneous infestations by different tick species in a single animal is rarely discussed in the literature. This study provides new insights into simultaneous infestations of different tick species on individual dogs in the Brazilian Amazon and reports, for the first time, the tick *A. latepunctatum* parasitizing a dog.

The literature documents up to three tick species infesting a single dog (Labruna et al., 2001; Costa et al., 2017). In the Amazon, there are records of five dogs being infested with *A. oblongoguttatum* and *A. ovale* in the municipality of Uruará, Pará state, and four dogs with *R. sanguineus* s.l. and *A. oblongoguttatum* in the municipality of Porto Velho (Labruna et al., 2000; Costa et al., 2021). Another study reported 11 tick species parasitizing dogs in Rondônia state; however, it did not discuss simultaneous infestations of these species (Labruna et al., 2005a).

In the Cerrado biome of the municipality of Franca (São Paulo state, Brazil), there are records of two dogs, each infested with two tick species: one with *Amblyomma sculptum* (previously published as *Amblyomma cajennense*) and *A. ovale*, and the other with *R. sanguineus* s.l. and *Rhipicephalus microplus* (Szabó et al., 2001). Rural dogs in an Amazon-Cerrado ecotone exhibited more simultaneous tick species infestations than urban dogs, likely due to their exposure to wild environments (Costa et al., 2017). In the present study, all dogs were reported by their owners to have free access to forested areas and exhibited simultaneous infestations of different species. The largest simultaneous infestation in a single dog involved six tick species and occurred in the MNP area (Table 1). Only dual infestations were observed in dogs from the NP area. The greater tick diversity in simultaneous infestations in dogs of the MNP area may be attributed to their access to larger forest fragments, where the diversity of wild hosts is likely greater than in the NP area, which is a smaller forest fragment with high human impact, which may have reduced the diversity of vertebrate hosts (Porto Velho, 2012; ICMBio, 2018).

Free-ranging dogs that come into contact with wild vegetation may be accidentally parasitized by ticks, and several tick species have been recorded on such dogs in the Brazilian Neotropical region, primarily from the genus *Amblyomma* (Labruna et al., 2000, 2005a). Seven tick species from two genera were reported parasitizing dogs in the present study: *R. sanguineus* s.l., *A. ovale*, *A. scalpturatum*, *A. latepunctatum*, *A. oblongoguttatum*, *A. naponense*, and *A. coelebs*. With the exception of *A. latepunctatum*, all the tick species identified here have been reported on dogs elsewhere. Among the tick species reported in dogs in the present study, *R. sanguineus* s.l. and *A. ovale* are particularly noteworthy for their significance in veterinary and public health. The dual infestation with these two tick species was the only one shared between the sites of this study.

*Rhipicephalus linnaei* is specific to parasitizing dogs in tropical regions worldwide (Slapeta et al., 2022), and although we have not identified what was the lineage of *R. sanguineus* s.l. of this study, is possible that the *Rhipicephalus* ticks of this study were *R. linnaei*, due to the region of the study is a tropical region, and independent of the *R. sanguineus* s.l. lineage, its occurrence was thus expected, especially in peri-urban areas and among domiciled dogs, as they are a known nidicolous tick (Linardi & Nagem, 1973; Ribeiro et al., 1997). Unrestrained dogs parasitized with *R. sanguineus* s.l. and with free access to wild environments may contribute to the disseminatation of this tick and also *Ehrlichia canis* to wild mammals (Iori et al., 1996; Labruna et al., 2005c; Santoro et al., 2016). There is evidence of *R. sanguineus* s.l. being infected by *Rickettsia rickettsii*, the causal agent of Brazilian spotted fever, and parasitizing a human, although this species is not considered a vector of *R. rickettsii* to humans (Cunha et al., 2009). These dynamics indicates that dogs may play a role in the circulation of pathogens from urban to sylvatic environments and vice versa. This highlights a bidirectional flow in this process, which could be amplified by the simultaneous parasitism by different tick species.

*Amblyomma ovale* occurs in the Neoarctic and Neotropical regions, from United States to Argentina, and parasitizes small rodents in the larvae and nymph stages, while when adults the species prefers carnivores (Guglielmone et al., 2003; Labruna et al., 2005a; Saraiva et al., 2012; Martins et al., 2016). Dogs are frequently infested with *A. ovale* in several regions of Brazil (Szabó et al., 2012; Costa et al., 2017; De Oliveira et al., 2019). This study corroborates previous reports that *A. ovale* is one of the most common ticks found in dogs and humans in the state of Rondônia (Labruna et al., 2005a).

In the present study, an engorged *A. ovale* female laid eggs, which confirms that dogs are suitable hosts for the adult stage of this tick species, as described previously (Martins et al., 2012). Although it is known to be an exophilic tick, previous studies have shown a surrogate life cycle of *A. ovale* in a peri-domiciliary environment, presenting nidicolous behavior. This phenomenon occurs when dogs were kept near to forested areas where *A. ovale* is present (Labruna, 2009; Szabó et al., 2009, 2012). Therefore, this behavior of *A. ovale* should be better investigated in the Amazon biome.

*Amblyomma ovale* is the main vector of *Rickettsia parkeri* strain Atlantic Forest to humans in the southern, southeastern and northeastern regions of Brazil and is the causal agent of *R. parkeri* spotted fever in the country (Spolidorio et al., 2010; Silva et al., 2011; Krawczak et al., 2016). The scenario of these diseases involves dogs with free access to forested areas (Szabó et al., 2012; Costa et al., 2017; De Oliveira et al., 2019). It is worth remembering that, although *A. ovale* has not been associated with the *R. parkeri* strain Atlantic Forest in the states of Rondônia and Amazonas, it has been reported in the neighboring state of Acre, which is also covered by the Amazon rainforest (Aguirre et al., 2022). Therefore, more studies need to be conducted with a One Health approach in order to confirm whether there is a *R*. *parkeri* spotted fever scenario in the Amazon at large.

*Amblyomma scalpturatum* and *A*. *latepunctatum* have been reported in South America, mainly in forested areas of the Amazon, thus being considered almost restricted to this area (Labruna et al., 2005a, b, 2010; Martins et al., 2014; Gianizella et al., 2018; Binetruy et al., 2019). The adult stages prefer to feed on large mammals, such as *Tapirus terrestris* and wild boars, while nymphs of these species may parasitize a wide range of small and large mammals (Jones et al.,1972; Labruna et al., 2002a, 2005b). Cases of parasitism in humans by *A. scalpturatum* have already been described in the literature however, to date, there are no records of human pathogens associated with this species of tick (Labruna et al., 2005a; Aguirre et al., 2019).

In the present study, nine partially engorged nymphs presented characteristics compatible with *A. scalpturatum* or *A. latepunctatum*. However, differentiation between these species was not possible due to the difficulty in visualizing the chitinous tubercles because of their stage of engorgement. Although all these ticks were considered genus *Amblyomma* in this work, the authors observed taxonomic differences between these species that do not appear in the dichotomous key, and they resemble nymphs of *A. scalpturatum*; the spurs of leg I in *A. scalpturatum* are more separate compared to those of *A. latepunctatum*. In addition, the cornuas of the dorsal base of the capitulum are more pronounced in *A. scalpturatum* than in *A. latepunctatum*. These characteristics may be relevant for the distinction between these species when engorged, which could become the subject of further studies. Although no nymph completed ecdysis, data from a collection conducted in the municipality of Candeias do Jamari, Rondônia, showed the successful parasitism of an *A. scalpturatum* nymph on a dog, suggesting that this mammal may act as suitable host for this species. Regarding the transmission of pathogens, *A. scalpturatum* has already been associated with the presence of DNA from *Rickettsia bellii* and *Rickettsia amblyommatis*, the latter of the spotted fever group, with pathogenicity that is still uncertain for humans (Labruna et al., 2004; Colle et al., 2020).

The distribution of *A. coelebs* is from Mexico to Argentina, and in Brazil it is found in the states of Acre, Amazonas, Mato Grosso, Mato Grosso do Sul, Pará, São Paulo, Espírito Santo, Roraima, Paraná, Minas Gerais, Bahia, Goiás and Rondônia (Guglielmone et al., 2003; Labruna et al., 2002b; Arzua et al., 2005; Martins et al., 2011; Saraiva et al., 2012 Gianizella et al., 2018; Rocha et al., 2021). The main host of this species is the tapir, although there are reports of parasitism in other animals, including dogs and humans (Labruna et al., 2005a; Garcia et al., 2015). This is the first report of *A. coelebs* larvae parasitizing dogs, but no ecdysis from larva to nymph or nymph to adult was observed in this study. However, there is a record in our laboratory of successful parasitism of *A. coelebs* nymph in a dog from complementary collections carried out in Candeias do Jamari, Rondônia (unpublished data), indicating the ability of dogs to serve as hosts for this species.

Cases of parasitism by *A. coelebs* in humans in Brazil have been reported mainly in the northern and midwestern regions, where the species is established (Labruna et al., 2004; Garcia et al., 2015; Suzin et al., 2022). Souza et al. (2022) also recorded *A. coelebs* with *R. amblyommatis* DNA. In this case, the patient was seropositive via indirect immunofluorescence for *Rickettsia* of the spotted fever group, although the patient did not present associated clinical manifestations.

*Amblyomma naponense* is distributed in South and Central America. In Brazil, it is found in the states of Amazonas, Acre, Pará, Paraná, Rondônia, Mato Grosso do Sul, Mato Grosso, Goiás, São Paulo, Minas Gerais, Bahia, Tocantins, Espírito Santo, Maranhão and Rio de Janeiro (Aragão, 1936; Guimarães et al., 2001; Labruna et al., 2002a, 2005a; Onofrio, 2007; Ogrzewalska et al., 2007; Costa et al., 2020)). It exhibits almost the same host preference, with a predilection for Tayassuidae in the adult stage, in addition to reports of parasitism in dogs and humans (Jones et al., 1972; Labruna et al., 2005a; Gianizella et al., 2018; Gruhn et al., 2019; Costa et al., 2020, 2021). The presence of *A. naponense* on dogs observed in the present study is in accordance with the existing literature. However, no engorged stages were observed that could suggest that dogs are good hosts for this species, and human parasitism was also not recorded. Additional unpublished data from the laboratory report an engorged *A. naponense* nymph on a dog in Candeias do Jamari, Rondônia, which was not able to perform adult ecdysis. Importantly, this species has already been associated with the presence of *R*. *bellii* DNA, a *Rickettsia* of the spotted fever group close to *Rickettsia africae*, and a *Rickettsia* of the Canadensis group (Soares et al., 2015).

*Amblyomma oblongoguttatum* is found in the Neotropical region, from Mexico to Brazil (Guglielmone et al., 2003). It parasitizes a wide range of small and large mammals in all stages, in addition to dogs and humans (Labruna et al., 2000, 2001, 2005a; Aragão, 1936; Freire, 1972; Martins et al., 2009; Costa et al., 2020, 2021). In the present study, five engorged *A. oblongoguttatum* females were observed; however, none of them were subjected to oviposition. In addition, three *A. oblongoguttatum* nymphs completed ecdysis to the adult stage (two females and one male). Based on these findings, we suggest that dogs may act as suitable hosts for *A. oblongoguttatum*, a hypothesis previously suggested by Labruna et al. (2000) in a study conducted in the Amazon region.

The exposure of dogs to wild environments can introduce zoonotic agents from the enzootic cycle and affect the health of wild animals by transmitting pathogens to animals that have no prior contact with these microorganisms. The opposite also occurs with the introduction of pathogens from the enzootic cycle to the zoonotic cycle, which can have direct consequences for human and animal health (Lindahl & Grace, 2015). Many zoonotic diseases, such as spotted fever, can be transmitted to humans, especially in areas where interactions between urban and wild environments are intense, with strong modification of landscapes (Thompson, 2013; Aguirre, 2017). This phenomenon occurs due to the overflow of agents that are originally contained in the silvatic context and is promoted by landscape modifications that facilitate and increase opportunistic and accidental relationships between parasites, hosts and the pathogen. This is inserted in the concept of One Health, which recognizes the interconnection between human health, animal health and environmental health (Thompson & Polley, 2014; Fiorello et al., 2017; Ellwanger & Chies, 2019).

The present study provides new information about simultaneous infestations of tick species on dogs, with a new report of quadruple, quintuple and sextuple infestations, thus presenting a specific profile of tick-dog interactions in the western Amazon region, and reports for the first time the presence of *A. latepunctatum* nymphal and *A. coelebs* larval infestations on dogs. This record exposes a potential for One Health in the Amazon region, as dogs can act as peri-domiciliary “hubs” of tick-borne pathogens that in natural conditions are not found in an animal with the ability to be simultaneously parasitized by these tick species, thereby increasing the chances of a spill over.
